# Differences between nitrogen-tolerant and nitrogen-susceptible sweetpotato cultivars in photosynthate distribution and transport under different nitrogen conditions

**DOI:** 10.1371/journal.pone.0194570

**Published:** 2018-03-29

**Authors:** Wenxue Duan, Qingmei Wang, Haiyan Zhang, Beitao Xie, Aixian Li, Fuyun Hou, Shunxu Dong, Baoqing Wang, Zhen Qin, Liming Zhang

**Affiliations:** 1 Crop Research Institute, Shandong Academy of Agricultural Sciences, Jinan, Shandong, China; 2 Scientific Observation and Experimental Station of Tuber and Root Crops in Huang-Huai-Hai Region, Jinan, Shandong, China; 3 Shandong Academy of Agricultural Sciences, Jinan, Shandong, China; Agriculture and Agri-Food Canada, CANADA

## Abstract

To characterize the differences in photosynthate distribution and transport between nitrogen(N)-tolerant and N-susceptible sweetpotato cultivars under different N conditions, three N levels, including 0 (N0), 120 (N120), and 240 kg ha^−1^ (N240), were used in field experiments with the Jishu26 (J26) and Xushu32 (X32) cultivars in 2015 and 2016. The results from both years revealed that high N application reduced the tuberous root yield, the tuber/vine rate of carbon-13 (^13^C), and top-to-base (three equal segments of stem divided from the fifth opened leaf of the shoot tip to the main stem, defined as the top, middle, and base parts, respectively) gradients such as sucrose, ammonia N and potassium along the stem. ‘J26’ showed a higher yield than ‘X32’ under N0 but lower yield than ‘X32’ under N120 and N240. It also exhibited a higher ^13^C distribution to tuberous roots compared with that of ‘X32’ under N0, and the opposite trend was observed under N120 and N240. Under N0, ‘J26’ showed a steep top-to-base amino acid gradient and a significantly lower top-to-base sucrose increase along the stem in the late growth stage. Under N120 and N240, ‘X32’ exhibited a greater top-to-base decrease in the ammonia N along the stem during the main growth stages, a steep top-to-base sucrose gradient along the stem in the early growth stage, and a lower top-to-base sucrose increase along the stem in the middle and late growth stages. The formation of a reasonable photosynthate distribution structure attributed to high yield was related to a desirable sucrose, ammonia N or K^+^ gradient downward along the stem. These results might help provide farmers with sweetpotato cultivars using less or no N fertilizer in soils of different fertility and enhance the knowledge of yield-related physiology.

## Introduction

Sweetpotato (*Ipomoea batatas*) is a versatile crop that is grown for its tubers. This crop produces high yields of root tubers per unit area and per unit time even in marginal lands [1,2]. Sweetpotato is largely produced in China, whose planting area and total production rank first worldwide [3]. Due to its wide adaptability, high yield and multiple uses, farmers can easily gain high planting benefits [4]. In the last decade, with the structural adjustment of agricultural system, sweetpotato is mainly planted not only in barren hills but also in fertile plain areas in northern China for higher planting benefits. To improve sweetpotato yield and increase the economic benefits, farmers not only choose more fertile soil but also focus more on the application of N fertilizer [5]. With high levels of N fertilizer, sweetpotato is easily overgrown even in moderately fertile soil. Sweetpotato plants have overgrown shoots under high N conditions, and excessive vine growth has been associated with lower storage root yield [6,7]. Previous studies have documented that N uptake and assimilation rates differ among sweetpotato cultivars, which can also exhibit differential N tolerance [8,9,10]. Hill et al. [10] identified several sweetpotato cultivars that could produce high yields without the addition of N fertilizer on N-deficient soils. Since most cultivars have overgrowth of vine and low yield in N-rich soils, it is necessary to identify cultivars with tolerance to high N conditions for farmers.

N is an essential element for growth and development and plays an important role in dry matter accumulation, phosphorus and potassium absorption, and root formation and enlargement [11,12,13]. N is also the main factor affecting the above-ground morphogenesis and root yield of sweetpotato [14,15,16,17]. Suitable N N application rates and methods are closely related to the soil and climatic conditions, cultivar types, and N fertilizer types. Phillips et al. [18] reported that optimum N application rates for sweetpotato varied annually under different precipitation conditions. According to Kaupa and Rao [19], co-application of mineral N and animal manure at a low dose of 50 kg N ha^-1^ improved sweetpotato tuber productivity in Papua New Guinea. Wu et al. [5] found that the shoot biomass of purple sweetpotato increased with the increase of N application under higher soil fertility. Studies of the relationships between N fertilizer and photosynthesis, growth, yield, and quality in sweetpotato have been reported previously [18,20,21,22]. However, differences in photosynthate distribution and transport in sweetpotato cultivars with different N tolerances under different N conditions have rarely been investigated.

Our previous results found the long-vine cultivar J26 and the short-vine cultivar X32 exhibited the highest yields under 30 kg N ha^-1^ and 90 N kg ha^-1^, respectively. In addition, these two cultivars showed similar yield potential in a field experiment in 2014 (S1 File). When N levels were increased to 120 kg ha^-1^, both cultivars showed yield reduction, and the root yield of ‘J26’ was significantly lower than that of ‘X32’. In the present study, we used these two cultivars in an experimental design involving a gradient of N fertilizer rates (0, 120 and 240 kg ha^-1^) to characterize their differences in photosynthate distribution in different organs using isotopic ^13^C labeling, distribution percentages of source-sink distance, sugar content in leaves, and sucrose, amino acid, and K^+^ gradients from the top to the base of the stem. The results may help farmers planting sweetpotato on soils of different fertility and increase our knowledge of yield-related physiology.

## Materials and methods

### Experimental site and plant materials

Field experiments were conducted in Changjia Village (36°79′N, 118°18′E), Licheng, Jinan, Shandong Province, China, from 2015 to 2016. The soil type was classified as sandy loam. The average annual temperature and rainfall in the study area are 13.0°C and 746.8 mm, respectively, with 60%–70% of rainfall occurring in the summer from June to August. The top 20 cm soil layer in the experimental field before sowing contained 12.9 g kg^-1^ of organic matter, 93.6 mg kg^-1^ of alkali-hydrolyzable N, 35.3 mg kg^-1^ of available phosphate, and 97.6 mg kg^-1^ of available potassium. Plants of two sweetpotato (*Ipomoea batatas* (L.) Lam.) cultivars, Jishu 26 (J26, a long-vine cultivar) and Xushu 32 (X32, a short-vine cultivar), were used in this study. Both of the orange-fleshed cultivars were suitable planted in northern China. We obtained the relevant permission from the corresponding institute (Crop Research Institute, Shandong Academy of Agricultural Sciences, China) for planting our materials in the field. We state that no specific permissions were required in the experimental site. We confirm that the field studies did not involve endangered or protected species.

### Experimental design

Three different N rates, including 0 (N0), 120 (N120), and 240 kg ha^−1^ (N240), were used in the field experiments with the Jishu26 (J26) and Xushu32 (X32) cultivars. The fertilizers contained urea (46.4% N), calcium triple superphosphate (46% P_2_O_5_), and potassium sulfate (50% K_2_O). Fixed amounts of P_2_O_5_ (75 kg ha^-1^) and K_2_O (150 kg ha^-1^) were added with varying amounts of N fertilizer as the basal fertilizer. Each treatment was replicated thrice in plots measuring 30 m^2^ (5 m × 6 m) using a completely randomized block design. All cultivars were planted on May 15, 2015, and May 13, 2016, with spacing of 80 cm × 24 cm (row width × plant spacing) and were harvested on October 24, 2015, and October 25, 2016, respectively. The other cultivation management practices were the same as in a typical field.

### Plant sampling

Five plants were randomly cut to collect above-ground organs at 50, 105, and 150 days after planting. The number of branches was determined within a length of 30 cm from the base of the main stem. The distance from the base of the fifth functional leaf to the base of the main stem was defined as the source–sink distance of the main stem. The source–sink distance of the remaining branches was measured, and the percentages of the different lengths (0–50 cm, 50–100 cm, 100–200 cm, and >200 cm) were calculated. Three equal segments of stem was divided from the fifth fully expanding leaf of the shoot tip to the main stem. The three equal parts were defined as the top, middle, and base parts, which were used as dry stem samples for determining the sucrose, free amino acid, and K^+^ contents. The fourth and fifth functional leaves of the main stem were taken as dried samples for determining the sucrose and starch contents. All of the dry samples were oven-dried at 70°C to a constant weight and then preserved in a desiccator for the following measurements.

### Variable measurements

The contents of sucrose and starch were determined by the sulfuric acid–anthrone colorimetric method according to Luo et al. [23]. The total free amino acid content was identified using the indene tri-ketone colorimetric method [24]. The K^+^ content was determined by a flame photometer as described by Chen et al. [25].

**Fig 1:**
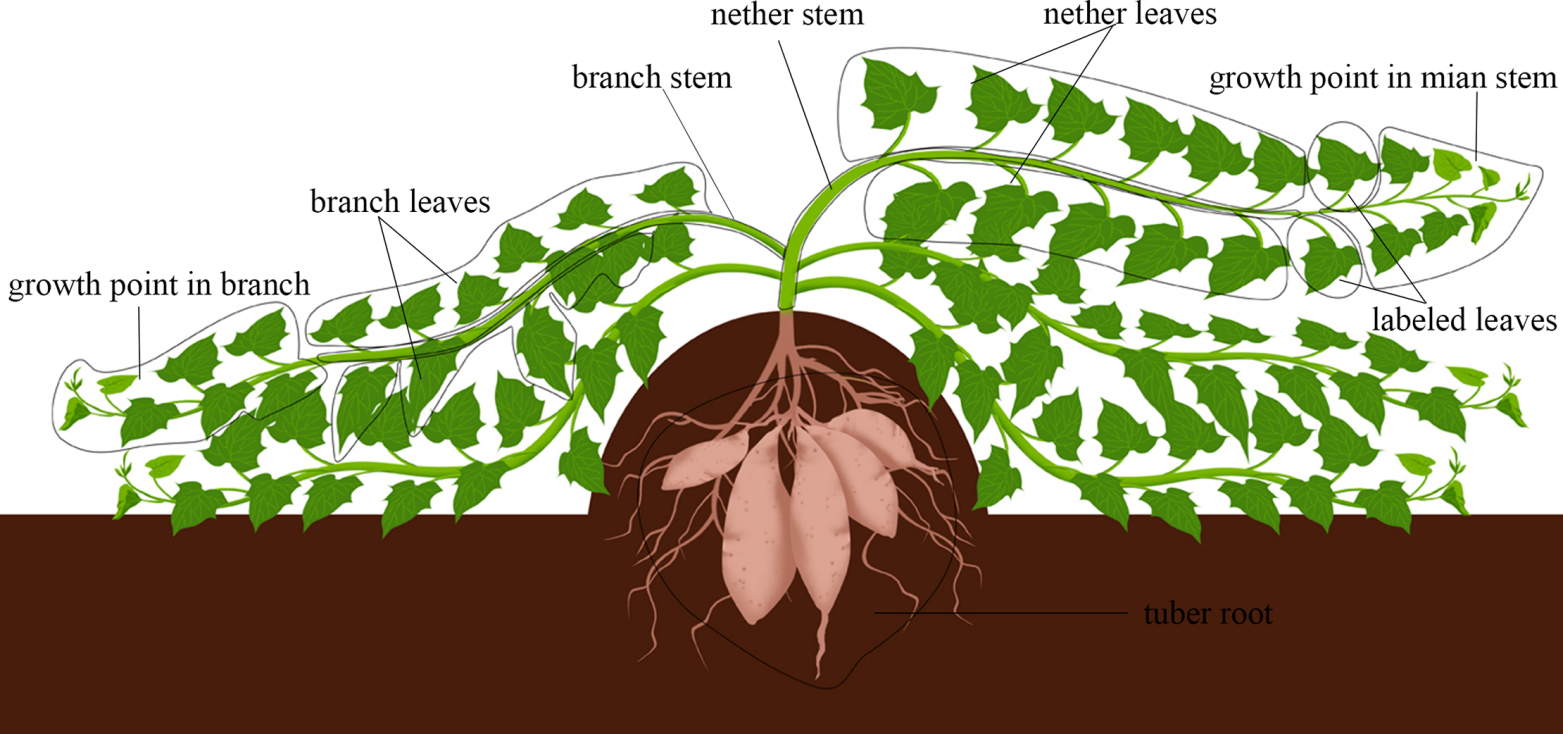
The description of ^13^C labeling sampling.

^13^C labeling of sweetpotato plants was performed at 55, 110, and 155 days after planting in 2016. At each labeling date, five plants were chosen, and the fourth or fifth fully expanding leaves from the shoot apex of the main stem were covered with an airbag. The volume of the airbag was 400 mL, and the leaves were suspended in the airbag. ^13^CO_2_ gas (50 mL, 8%) was injected into each airbag, and the volume of ^13^CO_2_ was approximately 1% of the total gas. ^13^CO_2_ (99% of atom ^13^C) was ordered from Shanghai Engineering Research Center of Stable Isotope. The plants were labeled for 30 min under the following growth conditions: radiant and enchanting sunshine, no wind or light wind, temperature of 25°C to 30°C, and relative humidity of 80% to 90%. After labeling, the airbags were removed. After 72 h, the plants were randomly uprooted to collect the storage roots and above-ground organs. The description of different organs for ^13^C labeling sampling was as shown in Fig 1. The labeled leaves of the main stem were defined as labeled leaves. The main stem was divided into two parts: the upper part was the part above the labeled leaves and was defined as the growing point of the main stem, whereas the nether part was under the labeled leaves. The lower part of the stem was designated the nether stem, and the unfolded leaves of the nether stem were designated the nether leaves. The remainder of the above-ground parts was named lateral branches. Each lateral branch was divided into two parts. The upper part was the part above the fifth fully expanded leaf from the shoot apex of the lateral branch and was defined as the growing point of the branch, whereas the nether part was under the fifth fully expanded leaf from the shoot apex. Moreover, this nether stem was designated the branch stem, and the leaves of this nether stem were designated the branch leaves. The stems were cut into horizontal sections approximately 5 cm in length. The storage roots were cut into pieces approximately 3 mm thick. The samples were dried using a DHG Series heating and drying oven (Model No. DHG-9203A, Shanghai Yiheng Technology, Co., Ltd., Shanghai, China). The leaves and petioles were collected, and stems and storage roots were cut, dried at 60°C in the oven and then ground to a powder using a Waring blender. The powder was analyzed using a stable isotope ratio mass spectrometer (IsoPrime 100, Elementar Analysensysteme GmbH Co. Ltd., Germany) [26].

The number of tubers per plant, weight of tubers per plant, and fresh yield of tubers were determined during the harvest period.

### Statistical analysis

Two-way ANOVA (cultivars and N amounts) and Duncan’s multiple range test were conducted using the statistical analysis software SPSS (version 13.0 for Windows, SPSS, Chicago, Illinois, USA). Each cultivar-N combination had three replicates.

## Results

### Root yield

The root yield of ‘J26’ under N0 in 2015 and 2016 was the highest, whereas the root yield under N240 was the lowest (Table 1). In 2015, the root yield of ‘X32’ under N0 and N120 was significantly higher than that under N240. ‘J26’ exhibited a significantly higher root yield than that of ‘X32’ under N0 but a significantly lower yield than that of ‘J26’ under N120 and N240 in both years. The percentage of yield reduction in ‘X32’ was lower than that in ‘J26’ under N120 and N240. In both cultivars, N240 resulted in the lowest number of tuber roots in 2016 and the lowest tuber root weight per plant in 2015 and 2016. The results indicated that abundant N supply caused yield reduction in both cultivars, ‘J26’ showed higher yield under low N conditions, and ‘X32’ showed a higher tolerance to N abundance.

**Table 1:** Number of tuber roots per plant, tuber root weight per plant and yield reduction under different treatments in 2015 and 2016.

Treatment	Number of tuber roots per plant	Tuber root weight per plant	Root yield	Percentage of yield reduction
		(g)	(kg ha^-1^)	(%)
**2015**				
**J26N0**	3.69bc	977.76a	50921.55a	
**J26N120**	3.31cd	793.03c	41301.13c	18.89
**J26N240**	2.90d	694.76d	36183.05d	28.94
**X32N0**	4.57a	907.28b	47251.30b	
**X32N120**	4.18ab	863.53b	44972.45b	4.82
**X32N240**	3.94b	789.65c	41125.21c	12.96
**2016**				
**J26N0**	2.75ab	781.65a	40708.54a	
**J26N120**	2.39bc	532.44d	27729.31d	31.88
**J26N240**	1.57d	328.29f	17097.31f	58.00
**X32N0**	3.19a	706.72b	36805.74b	
**X32N120**	2.75ab	604.98c	31507.10c	14.40
**X32N240**	1.97cd	424.70e	22118.17d	39.91

Values within the same column of the same year followed by the same letter are not significantly different at the 5% level by ANOVA-Duncan’s multiple range test.

### Sucrose content, starch content, and sucrose/starch ratio in the leaves

The starch content in the leaves of ‘J26’ was the highest, whereas the sucrose/starch ratio in the leaves was the lowest under N240 at 50th day after planting in both years (Table 2). ‘J26 exhibited significantly lower starch content in the leaves under N0 compared with that of ‘X32’ in both years. Under N120 and N240, ‘X32’ showed significantly higher sucrose content in the leaves than that of ‘J26’ in 2016 and a significantly higher sucrose/starch ratio in the leaves than that of ‘J26’ in both years. The sucrose content and sucrose/starch ratio in the leaves of ‘J26’ under N0 were the highest, whereas those under N240 were the lowest at 105th day after planting in both years. ‘J26’ exhibited significantly higher sucrose content and sucrose/starch ratio in the leaves than those of ‘X32’ under N0 in 2015. Under N120 and N240, ‘X32’ exhibited a significantly higher sucrose/starch ratio in the leaves than that of ‘J26’ in both years. N240 caused the lowest sucrose content in the leaves of ‘J26’ and the lowest sucrose/starch ratio in the leaves of both cultivars at 150th day after planting in both years. ‘J26’ under N0 exhibited significantly higher sucrose content and sucrose/starch ratio in the leaves than those of ‘X32’ in 2016, and ‘X32’ exhibited a significantly higher sucrose/starch ratio in the leaves than that of ‘J26’ under N120 and N240 in both years.

**Table 2:** Sucrose content, starch content and sucrose/starch ratio in leaves during the main growth stages under different treatments in 2015 and 2016.

	50 d after planting			105 d after planting			150 d after planting		
Treatment	Sucrose content	Starch content	Sucrose/Starch	Sucrose content	Starch content	Sucrose/ Starch	Sucrose content	Starch content	Sucrose/ Starch
	(%)	(%)		(%)	(%)		(%)	(%)	
**2015**									
**J26N0**	2.84cd	10.16c	0.28a	4.79a	15.54a	0.31a	3.69a	13.32b	0.28a
**J26N120**	2.53d	11.27c	0.22c	3.65b	15.84a	0.23d	3.35a	14.14ab	0.24b
**J26N240**	3.20c	18.45a	0.17d	2.86cd	14.06b	0.20e	2.47b	14.10ab	0.18c
**X32N0**	4.71a	17.19ab	0.27ab	2.68d	10.37c	0.26b	3.89a	13.53b	0.29a
**X32N120**	4.08b	15.86b	0.26b	2.77d	10.95c	0.25bc	4.09a	15.00ab	0.27a
**X32N240**	3.18c	15.52b	0.20c	3.24bc	13.72b	0.24cd	3.65a	15.41a	0.24b
**2016**									
**J26N0**	2.40c	10.72d	0.22b	3.84b	13.84c	0.28a	5.86a	21.55ab	0.27a
**J26N120**	2.64c	15.08bc	0.18c	2.90c	14.18c	0.20c	4.33b	23.00a	0.19c
**J26N240**	2.19c	16.89a	0.13d	2.17d	12.89c	0.17d	3.09d	19.17c	0.16d
**X32N0**	3.42b	14.01c	0.24ab	5.00a	18.56ab	0.27ab	4.78b	20.38bc	0.23b
**X32N120**	4.17a	16.09ab	0.26a	4.91a	19.56a	0.25b	4.74b	21.09abc	0.22b
**X32N240**	4.29a	16.95a	0.25a	3.80b	17.56b	0.22c	3.74c	19.63bc	0.19c

Values within the same column of the same year followed by the same letter are not significantly different at the 5% level by ANOVA-Duncan’s multiple range test.

These data indicated that abundant N supply reduced the sucrose content and sucrose/starch ratio in the leaves in the middle and late growth stages in both cultivars. ‘J26’ exhibited a higher sucrose/starch ratio in the leaves in the middle growth stage in 2015 and in the late growth stage in 2016 under low N conditions, and ‘X32’ exhibited a higher sucrose/starch ratio in the leaves under high N conditions during the main growth stages.

### Source–sink distance and its distribution percentage

**Fig 2:**
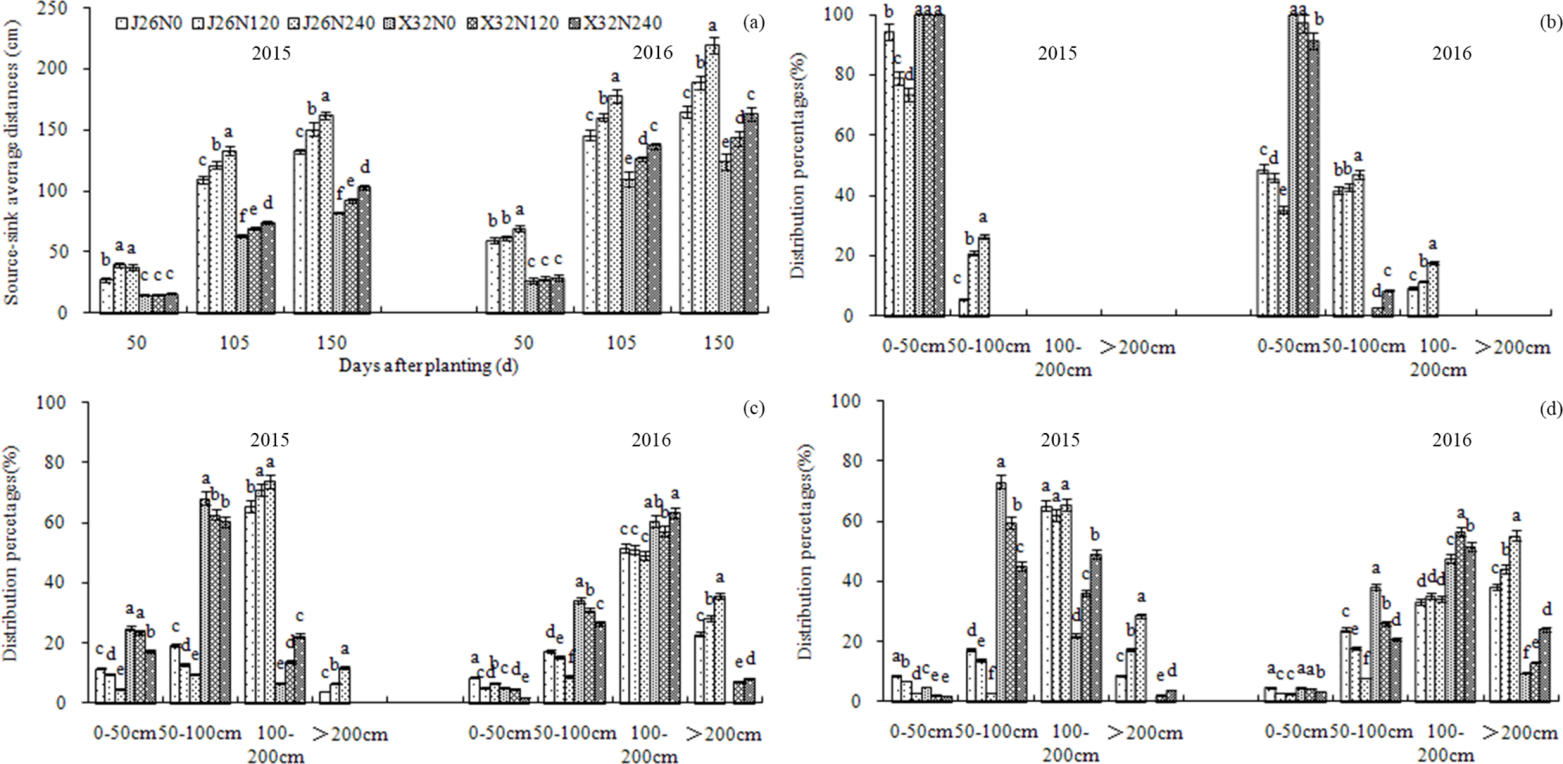
**Source-sink average distances at the main grwoth stages (a) and distribution percentages of different source-sink distances at 50 d after planting (b), at 105 d after planting (c) and at 150 d after planting (d) under different treatments in 2015 and 2016.** Error bars represent standard errors of the means (P<0.05).

Compared with ‘J26’, ‘X32’ had a significantly lower source–sink average distance under the same N conditions (Fig 2A). N240 significantly increased the source–sink average distance in ‘J26’ at 50th day after planting in both years. The source–sink average distance was the highest under N240 and the lowest under N0 in both cultivars at 105th day after planting in 2015 and 2016 (Fig 2A). N120 and N240 significantly increased the distribution percentages at >200 cm in ‘J26’ in both years (Fig 2C). In ‘X32’, N120 and N240 significantly decreased the distribution percentages at 50–100 cm in 2015 and 2016 and increased those at 100–200 cm in 2015 and at >200 cm in 2016. Compared with ‘J26’, ‘X32’ had significantly lower distribution percentages at >200 cm and significantly higher distribution percentages at 50–100 cm under the same N conditions. The changes in the source–sink distance and its distribution percentage at 150th day after planting showed trends similar to those at 105th day after planting (Fig 2A, 2C and 2D).

These results indicated that abundant N supply increased the source–sink average distance in both cultivars. In the middle and late growth stages, high N levels significantly increased the excessively long source–sink distance distribution percentages at >200 cm in ‘J26’ and at 100–200 cm and >200 cm in ‘X32’, respectively. Compared with ‘J26’, the short-vine cultivar X32 exhibited significantly higher source–sink distance distribution percentages at 50–100 cm and lower distribution percentages at >200 cm in the middle and late growth stages under the same N conditions.

### ^13^C distribution

**Fig 3:**
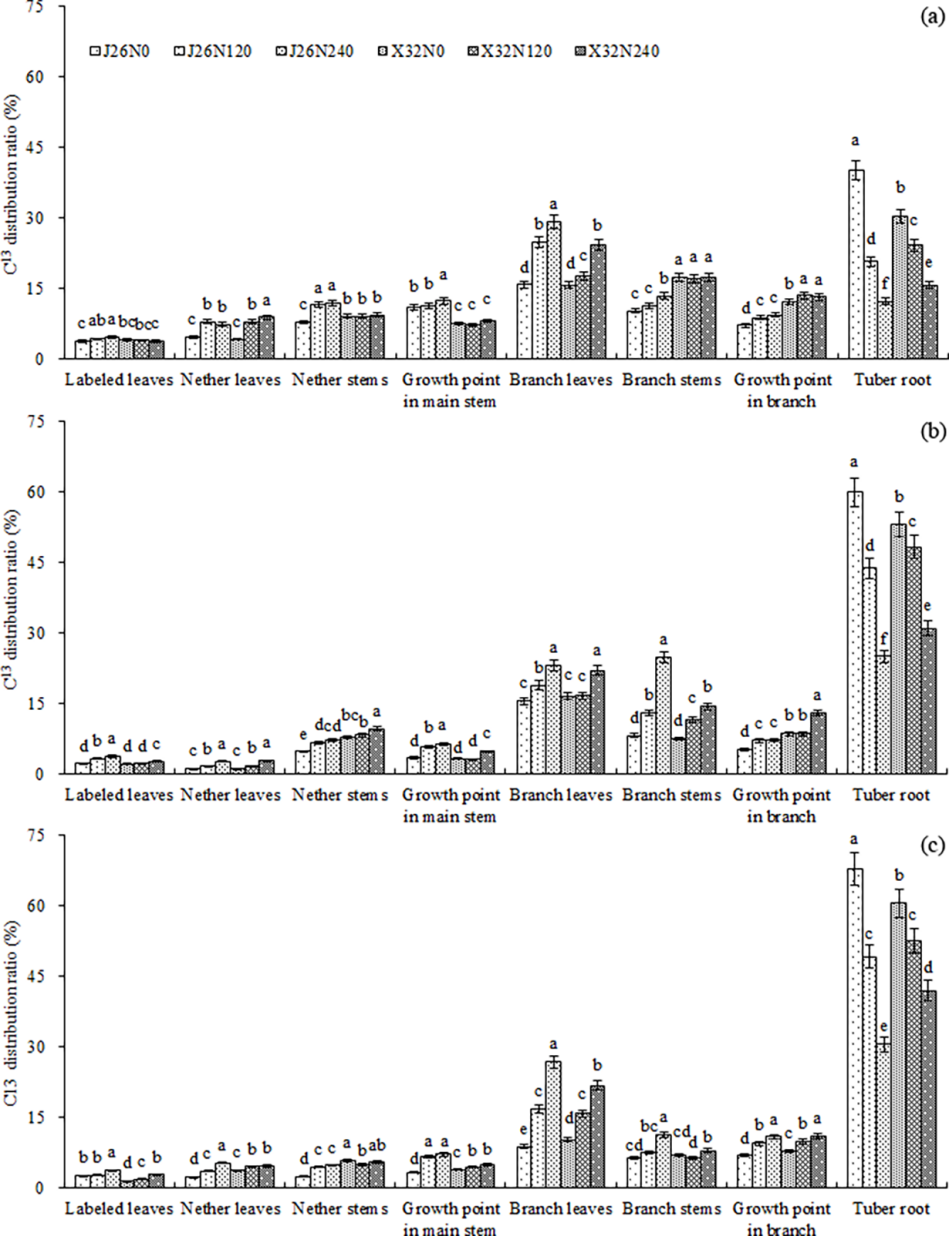
^**13**^**C distribution ratio in different organs at 55 d after planting (a), at 110 d after planting (b) and at 155 d after planting (c) under different treatments in 2016.** Error bars represent standard errors of the means (P<0.05).

The ^13^C distribution ratio in tuberous roots was the lowest under N240 and the highest under N0 in both cultivars during the main growth stages in 2016 (Fig 3). In the above-ground parts, ^13^C was mainly distributed in branch leaves, branch stems, and the growth point in the branch at 55th day after planting (Fig 3A). N120 and N240 significantly increased the ^13^C distribution ratio in the nether stems and the growth point in the branch in ‘J26’ and the branch leaves and the growth point in the branch in ‘X32’. Moreover, the ^13^C distribution ratio at the growth point in the main stem, branch leaves, and branch stems under N240 in ‘J26’ was the highest. The ^13^C distribution ratio in the tuberous roots of ‘J26’ was significantly higher than that of ‘X32’ under N0 and significantly lower than that of ‘X32’ under N120 and N240 (Fig 3A). ^13^C was mainly distributed in the branch leaves, branch stems, and the growth point in the branch in the above-ground parts at 110th day and 155th day after planting (Fig 3B and 3C). N240 significantly increased the ^13^C distribution ratio in the branch leaves and branch stems in ‘J26’ and the branch stem, branch leaves, and growth point in the branch in ‘X32’ at 110th day after planting (Fig 3B). For both cultivars, the ^13^C distribution ratio in the branch leaves, branch stems, and growth point in the branch under N240 was the highest at 155th day after planting (Fig 3C). The ^13^C distribution ratio in the tuberous roots at 110th day after planting showed trends similar to those at 55th day after planting. ‘J26’ showed a significantly higher ^13^C distribution ratio in the tuberous roots than that of ‘X32’ under N0 but a significantly lower ^13^C distribution ratio in the tuberous roots than that of ‘J26’ under N240 at 155th day after planting (Fig 3C).

These results indicated that abundant N supply decreased the ^13^C distribution ratio in the tuberous roots of both cultivars because of increasing ^13^C allocation to the above-ground parts, especially to the branch leaves, branch stems, and growth point in the branch. Comparing the cultivars, ‘J26’ exhibited a lower ^13^C distribution ratio in the above-ground parts and a higher ^13^C distribution ratio in tuberous roots under low N conditions, while ‘X32’ exhibited an opposite change trend under high N conditions.

### Sucrose content in the stem

The sucrose content from the top to the base of the stem in both cultivars decreased under N0 and N120 at 50th day after planting in both years. An opposite trend was observed under N240 in ‘J26’ in 2015 and 2016 and in ‘X32’ in 2016 (Table 3). N240 exhibited the highest sucrose content at the base of the stem in both cultivars in 2015. ‘J26’ showed a greater decrease in the top-to-base sucrose content along the stem under N0 compared with that of ‘X32’ in 2016, while ‘X32’ under N120 showed an opposite trend compared with ‘J26’ in both years. The sucrose content at the base of the stem in both cultivars under N240 was the highest and that under N0 was the lowest at 105th day after planting in both years. The sucrose content from the top to the base of the stem in both cultivars showed an increasing trend under N240 in 2015 and 2016, and this phenomenon also occurred under N120 in ‘J26’ in 2016. The decrease in the amplitude of sucrose content from the top to the base of the stem was the greatest under N0 in ‘J26’ in 2015 and in both cultivars in 2016. ‘J26’ showed a significantly lower sucrose content at the base of the stem and a greater decrease in top-to-base sucrose content under N0 compared with that of ‘X32’ in 2015. ‘X32’ exhibited a significantly lower increase in the amplitude of sucrose content from the top to the base of the stem under N240 than that of ‘J26’ did in both years. The sucrose content at the base of the stem was higher than that at the top of the stem in all treatments at 150th day after planting. The sucrose content was the highest at the base of the stem in both cultivars under N240 and the lowest under N0 in both years. N120 and N240 caused a greater increase in the top-to-base sucrose content along the stem in ‘J26’ in both years. In ‘X32’, N240 caused a significant increase in the amplitude of sucrose content from the top to the base of the stem. ‘J26’ exhibited a significantly lower increase in the amplitude of sucrose content from the top to the base of the stem under N0 compared with that of ‘X32’, while an opposite change trend was observed under N120 and N240 in both years.

**Table 3:** Sucrose content at the top of the stem and at the base of the stem, and its gradient from the top to base of the stem during the main growth stages under different treatments in 2015 and 2016.

	50 d after planting			105 d after planting			150 d after planting		
Treatment	Top	Base	Top-base	Top	Base	Top-base	Top	Base	Top-base
	(%)	(%)	(%)	(%)	(%)	(%)	(%)	(%)	(%)
**2015**									
**J26N0**	5.40bc	4.35c	24.03a	6.72c	5.10d	31.65a	5.00b	5.52cd	-9.34a
**J26N120**	5.64b	4.95b	13.91c	6.73c	6.57b	2.40c	4.16c	5.28d	-21.09c
**J26N240**	6.21a	6.44a	-3.57e	6.94c	9.77a	-29.01e	4.95b	7.39b	-33.05e
**X32N0**	4.85c	3.96c	22.72ab	7.34c	5.82c	26.19b	5.09b	5.81c	-12.47b
**X32N120**	5.18bc	4.26c	21.49b	8.06b	6.40b	25.96b	6.59a	7.69b	-14.31b
**X32N240**	6.17a	6.02a	2.51d	8.97a	9.38a	-4.39d	6.33a	8.46a	-25.15d
**2016**									
**J26N0**	5.46c	4.17b	30.94a	7.71b	6.17d	24.89a	4.98a	5.96cd	-16.35a
**J26N120**	5.73c	5.55a	3.18d	5.76c	5.94d	-3.01c	5.64a	7.43b	-24.05c
**J26N240**	5.68c	5.82a	-2.39e	4.84d	7.76b	-37.63e	5.48a	8.46a	-35.14e
**X32N0**	6.78a	5.36a	26.54b	7.39b	6.00d	23.10a	4.21b	5.20d	-19.14b
**X32N120**	6.27b	5.27a	18.89c	8.46a	7.14c	18.42b	5.04a	6.24c	-19.22b
**X32N240**	5.64c	5.78a	-2.42e	8.42a	8.97a	-6.17d	5.52a	7.83ab	-29.51d

Values within the same column of the same year followed by the same letter are not significantly different at the 5% level by ANOVA-Duncan’s multiple range test. A positive gradient means sucrose content increasing from the top to base of the stem, whereas a negative gradient means sucrose content decreasing from the top to base of the stem.

These results indicated that abundant N supply increased the sucrose content at the base of the stem, lessened the decrease in the amplitude of sucrose content from the top to the base of the stem in the early and middle growth stages, and caused a greater top-to-base sucrose content increase in the late growth stage. Comparing the cultivars, ‘J26’ showed a significantly lower increase in the amplitude of sucrose content from the top to the base of the stem under N0 in the late growth stage. ‘X32’ exhibited a significantly greater decrease in the top-to-base sucrose content under N120 in the early and middle growth stages and a lower increase in the top-to-base sucrose content along the stem under N240 in the middle and late growth stages.

### Free amino acid content in the stem

For both cultivars, the free amino acid content decreased from the top to the base of the stem during the main growth stages in both years (Table 4). The decrease in the amplitude of the amino acid content from the top to the base of the stem under N120 and N240 was significantly less than that under N0 in ‘J26’ at 50th day after planting in both years. ‘J26’ exhibited a significantly greater decrease in the amplitude of amino acid content from the top to the base of the stem under N0 than that of ‘X32’ in 2016, while ‘X32’ showed a significantly greater decrease in the amplitude of amino acid content under N120 and N240 than that of ‘J26’ in both years. The decrease in the amplitude of amino acid content from the top to the base of the stem under N0 was the highest and that under N240 was the lowest in both cultivars at 105th day after planting in both years. Compared with ‘J26’, ‘X32’ showed a significantly greater decrease in the amplitude of amino acid content from the top to the base of the stem under N120 and N240 in 2015 and under N240 in 2016, respectively. The decrease in the amplitude of amino acid content from the top to the base of the stem under N0 was the highest and that under N240 was the lowest in ‘J26’ at 150th day after planting in both years. ‘J26’ exhibited a significantly greater decrease in the amplitude of amino acid content from the top to the base of the stem under N0 than that of ‘X32’ in 2015 and 2016, while an opposite trend was observed under N120 and N240 in ‘X32’.

**Table 4:** Free amino acid content at the top and base of the stem and the gradient from the top to base of the stem during the main growth stages under different treatments in 2015 and 2016.

	50 d after planting			105 d after planting			150 d after planting		
Treatment	Top	Base	Top-base	Top	Base	Top-base	Top	Base	Top-base
	(%)	(%)	(%)	(%)	(%)	(%)	(%)	(%)	(%)
**2015**									
**J26N0**	194.16cd	113.81d	70.61a	165.22d	93.20c	77.27a	129.63d	82.16d	57.79a
**J26N120**	221.07bc	148.47bc	48.90c	214.11c	149.69b	43.04c	142.51cd	102.42cd	39.15c
**J26N240**	248.21b	189.53a	30.96d	240.04bc	182.25ab	31.71d	151.36cd	120.01bc	26.13d
**X32N0**	173.69d	103.05d	68.55ab	171.16d	97.52c	75.52a	162.60bc	109.09bc	49.06b
**X32N120**	213.05bc	127.36cd	67.28ab	258.34ab	172.11ab	50.10b	185.18ab	125.90b	47.09b
**X32N240**	285.02a	172.21ab	65.51b	274.30a	194.42a	41.08c	205.61a	147.96a	38.97c
**2016**									
**J26N0**	171.63c	96.74e	77.40a	173.76d	114.29d	52.04a	143.13d	88.92c	60.96a
**J26N120**	237.75b	156.71c	51.71d	247.98b	168.26c	47.38b	160.30d	116.37b	37.75d
**J26N240**	290.29a	225.53a	28.71f	294.19a	239.63a	22.77e	188.52b	159.45a	18.23f
**X32N0**	220.84b	132.19d	67.06b	206.89c	138.81d	49.05a	163.63cd	110.81b	47.66b
**X32N120**	279.25a	175.95bc	58.71c	279.49a	195.75b	42.78c	186.92bc	129.70b	44.12b
**X32N240**	287.39a	197.85b	45.26e	296.21a	217.50ab	36.19d	213.86a	173.94a	22.95e

Values within the same column of the same year followed by the same letter are not significantly different at the 5% level by ANOVA-Duncan’s multiple range test.

These results indicated that abundant N supply reduced the decrease in the amplitude of amino acid content from the top to the base of the stem in both cultivars during the main growth stages. ‘J26’ showed a significantly higher decrease in the amplitude of amino acid content from the top to the base of the stem under N0 in the late growth stage, while ‘X32’ exhibited a greater decrease in the amplitude of amino acid content under N120 and N240 during the main growth stages.

### K^+^ content in the stem

At 50th days after planting, the decrease in the amplitude of K^+^ content from the top to the base of the stem under N240 was the lowest in ‘J26’ in 2015 and 2016 and in ‘X32’ in 2016 (Table 5). ‘J26’ exhibited a significantly lower K^+^ content at the base of the stem and a greater decrease in the amplitude of K^+^ content from the top to the base of the stem under N0 compared with those of ‘X32’ in 2015. Compared with ‘J26’, the amplitude of K^+^ content in ‘X32’ decreased to a greater extent from the top to the base of the stem under N240 in 2016. The decrease in the amplitude of K^+^ content from the top to the base of the stem in ‘J26’ was the lowest under N240 and the highest under N0 at 105th day and 150th day after planting in both years. In ‘X32’, the decrease in the amplitude of K^+^ content under N0 did not differ from that under N120 but was significantly greater than that under N240. ‘J26’ exhibited a significantly lower K^+^ content at the base of the stem and a greater decrease in the amplitude of K^+^ content from the top to the base of the stem under N0 compared with those of ‘X32’ at 105th day after planting in both years. ‘X32’ showed a significantly greater decrease in the amplitude of K^+^ content than that of ‘J26’ under N120 and N240 in 2015 and under N240 in 2016. Compared with ‘J26’, ‘X32’ showed a significantly greater decrease in the amplitude of K^+^ content from the top to the base of the stem under N240 in 2015 and under N120 and N240 in 2016 at 150th day after planting.

**Table 5:** K^+^ content at the top and base of the stem and the gradient from the top to base of the stem during the main growth stages under different treatments in 2015 and 2016.

	50 d after planting			105 d after planting			150 d after planting		
Treatment	Top	Base	Top-base	Top	Base	Top-base	Top	Base	Top-base
	(%)	(%)	(%)	(%)	(%)	(%)	(%)	(%)	(%)
**2015**									
**J26N0**	15.71c	9.55d	64.49a	16.54d	10.16c	62.76a	14.48d	9.06d	59.86a
**J26N120**	21.09ab	13.09ab	61.15b	17.97c	11.93b	50.65c	16.20c	10.87c	49.10c
**J26N240**	21.61a	13.97a	54.69c	17.15cd	12.08b	41.98d	16.95c	11.75bc	44.30d
**X32N0**	16.79c	10.52c	59.58b	19.21b	12.25b	56.75b	17.31c	11.06c	56.59ab
**X32N120**	16.29c	10.28c	58.49b	20.68a	13.26a	55.98b	19.22b	12.53b	53.38bc
**X32N240**	19.62b	12.53b	56.60bc	20.48a	13.66a	49.89c	21.62a	14.37a	50.44c
**2016**									
**J26N0**	17.48c	11.21c	55.95ab	17.73d	11.01d	60.96a	15.56d	9.64d	61.39a
**J26N120**	22.31a	14.51a	53.69bc	19.34cd	12.88c	50.22c	16.53d	11.02c	50.02c
**J26N240**	21.69ab	14.70a	47.53d	20.37c	14.08bc	44.63e	18.90c	13.31b	41.93e
**X32N0**	16.63c	10.55c	57.56a	21.06bc	13.64bc	54.45b	20.33bc	12.69b	60.19ab
**X32N120**	20.20b	12.92b	56.29ab	22.61ab	14.69b	53.89bc	21.28ab	13.56b	56.97b
**X32N240**	23.18a	15.30a	51.56c	23.67a	15.96a	48.25d	22.45a	15.45a	45.32d

Values within the same column of the same year followed by the same letter are not significantly different at the 5% level by ANOVA-Duncan’s multiple range test.

These results indicated that abundant N supply decreased the amplitude of K^+^ content from the top to the base of the stem in ‘J26’ during the main growth stages, and N240 decreased that in ‘X32’ in the middle and late growth stages. ‘J26’ exhibited a significantly higher decrease in the amplitude of K^+^ content from the top to the base of the stem under low N conditions in the middle growth stage, while ‘X32’ exhibited a significantly higher decrease in the amplitude of K^+^ content under high N conditions in the middle and late growth stages.

## Discussion

A wide range of N fertilizer requirements has been reported for sweetpotato, but much depends on the cultivar, soil type and climatic conditions [5,18,19,27]. According to Hartemink et al. [27], sweetpotato root yield is negatively affected by N fertilizers. Wu et al. [5] reported that the root yield of cultivar Zijing No. 2 decreased when N application was increased to 75 kg ha^–1^ in fertile soil. Based on a former field experiment in 2014, 120 kg N ha^–1^ caused toxic effects on root yield in both ‘J26’ and ‘X32’, which had similar yield potentials under suitable N conditions. In the present study, a gradient of N fertilizer rates (0, 120, and 240 kg ha^–1^) was designed and the responsiveness to high N was verified in each cultivar. Thus, the yield of both cultivars significantly decreased because of high N application. Genotypic differences in sweetpotato cultivars could influence N uptake and dry matter partitioning [8]. Wilson [28] classified sweetpotato cultivars into N-responsive, N-indifferent, and N-depressive types. Wu et al. [5] reported that cultivars with high N demand had higher root yield in fertile soils. Xu et al. [29] found that the ^15^N accumulation and distribution ratios in the tuber roots of a short-vine cultivar were higher than those of a long-vine cultivar, and the short-vine cultivar exhibited higher root yield under high N conditions. In this study, ‘J26’ had the highest yield and exhibited a significantly higher yield than that of ‘X32’ under low N conditions. This effect might be attributed to the relatively lower N demand of ‘J26’. Under high N conditions, ‘J26’ showed serious yield reduction and exhibited a significantly lower yield than that of ‘X32’. This finding might be attributed to the superfluous accumulation of N in the above-ground parts of ‘J26’, which could easily cause vine overgrowth and source–sink imbalance, reducing yield. However, in ‘X32’, this effect might be attributed to high N demand and more N accumulation and distribution in tuberous roots than in the above-ground parts, which were beneficial for the coordinated growth of vines and roots, improving root yield. These differences in N utilization by different sweetpotato cultivars suggested that excessive shoot growth and poor yield due to excessive N by over-fertilization or high soil fertility could potentially be overcome by cultivars with high tolerance to abundant N. For practical production of sweetpotato, farmers can not only gain higher output but also require less or no N fertilizer by using cultivar J26 in soils with fertility similar to that in this study. Farmers planting sweetpotato in more fertile soils should either use no N application or use cultivar X32, which has a high tolerance to abundant N.

A constant sucrose concentration, together with an increased capacity for sucrose synthesis in source tissues, can indicate a potentially elevated flux of carbohydrates from source to sink [30]. A linear relationship between sucrose content and export rate in plant leaves has been reported [31,32,33]. Jonik et al. [34] found that triple-transgenic potato plants rerouted photoassimilates to sink organs at the expense of leaf starch accumulation. In cotton, the decreased sucrose/starch ratio in the subtending leaves of cotton bolls was associated with the low exportation of sucrose [35]. A higher sucrose/starch ratio was beneficial for sucrose transport from leaves. In this study, abundant N supply caused lower sucrose/starch ratio in the leaves in the middle and late growth stages. Comparing the cultivars, ‘J26’ exhibited a higher sucrose/starch ratio in the leaves in the middle growth stage in 2015 and in the late growth stage in 2016 under low N conditions, whereas ‘X32’ exhibited a higher sucrose/starch ratio in the leaves during the main growth stages under high N conditions. This higher ratio was beneficial for improving the ratio of transportable sucrose; thus, increasing sucrose transport from leaves.

The cultivars display different storage root yield potentials, which can be attributed to the rate of photosynthesis, the efficiency of assimilate partitioning to the storage roots, and the maturity period [2,36,37]. The timely and efficient distribution of assimilates to tuberous roots is the basis for high yield in sweetpotato [38], and the storage roots are formed and developed faster if assimilates are transported to roots earlier [39]. Compared with those of low-yielding sweetpotato cultivars, the storage roots of high-yielding sweetpotato cultivars became photosynthate supply centers (^13^C distribution ratio ≥50%) earlier, and photosynthate consumption in the above-ground parts was reduced [26]. The study found that the ^13^C in the above-ground parts was mainly distributed in the branch leaves, branch stems, and the growth point in the branch. High N application caused the above-ground parts to become the ^13^C supply center and, thus, reduced the ^13^C distribution ratio in tuberous roots. ‘J26’ exhibited a desirable distribution structure of photosynthates and distributed more photosynthates into the tuberous roots than ‘X32’ under low N conditions, leading to a higher storage root yield. Under high N conditions, ‘X32’ consumed fewer photosynthates for the growth of the above-ground parts and promoted more photosynthate accumulation in tuberous roots compared with that of ‘J26’, thus gaining a higher root yield. In summary, a reasonable distribution pattern of photosynthates that promotes the timely transformation of the growth center from the above-ground parts to the tuberous roots need to be established.

The source-to-sink transport of sugars is one of the major determinants of plant growth, and this mechanism relies on the efficient and controlled distribution of sucrose among plant organs through the phloem [40]. In the castor bean, the sucrose content was the highest at the uppermost internodes and gradually decreased from the top to the base of the stem [41]. The sucrose content gradually declined along the axis in potato [42], whereas an increasing tip-to-base sucrose gradient was found in the phloem apoplast of *Phaseolus* [43]. In this study, the sucrose content declined from the top to the base of the stem in the early growth stage and increased in the middle and late growth stages in sweetpotato. Higher sucrose content at the base of the stem caused by high N application indicated that the sucrose unloading in the sink might be inhibited. The most widely accepted concept to explain solute transport in the phloem is mass flow, as initially proposed by Münch [40,44,45,46,47,48]. According to the Münch hypothesis, the solution flow through the phloem is driven by a hydrostatic pressure gradient [49]. Together with potassium, sugars and amino N compounds are the principal osmotic components. Thus, they influence the rates of phloem transport and assimilate partitioning patterns [40,48]. The concentration gradient must be as steep as possible to achieve maximal source–sink transfer [50]. In this study, the plants at high N rates of 120 kg ha^–1^ or 240 kg ha^–1^ exhibited lower decreases in the amplitude of amino acid or K^+^ content downward along the stem in the middle and late growth stages, indicating that abundant N supply had deleterious effects on the generation of the hydrostatic pressure gradient. Compared with ‘X32’, ‘J26’ showed a steep top-to-base K^+^ gradient in the middle growth stage and a steep top-to-base amino acid gradient in the late growth stage under low N conditions. However, under high N conditions, ‘X32’ showed a greater decrease in the amplitude of amino acid content and K^+^ content from the top to the base of the stem and exhibited a desirable top-to-base sucrose gradient in the middle and late growth stages. A steep amino acid or K^+^ content gradient was beneficial for generating a hydrostatic pressure gradient and correlated with a desirable sucrose gradient for its transport downward along the stem, so that more photosynthates were transported to the tuberous roots, as shown in the ^13^C labeling tests, and a higher root yield was achieved.

## Conclusions

Increasing N rates to 120 kg ha^−1^ or 240 kg ha^−1^ resulted in decreased top-to-base amino acid and K^+^ gradients and an undesirable top-to-base sucrose gradient along the stem. High N supply also decreased the ^13^C distribution ratio in tuberous roots. ‘J26’ showed a steep top-to-base K^+^ gradient along the stem in the middle growth stage and a steep top-to-base amino acid gradient along the stem in the late growth stage under low N conditions. Under high N conditions, ‘X32’ exhibited a greater decrease in the amplitude of ammonia N and K^+^ content from the top to the base of the stem in the middle and late growth stages, a steep top-to-base sucrose gradient along the stem in the early growth stage, and a lower increasing amplitude of sucrose content downward along the stem in the middle and late growth stages. The ^13^C distribution ratio in the tuberous roots of ‘J26’ was significantly higher than that of ‘X32’ under low N conditions and an opposite trend was observed under high N conditions. The formation of a reasonable photosynthate distribution structure attributed to high yield was related to a desirable sucrose, ammonia N, or K^+^ gradient downward along the stem. For the practical production of sweetpotato, farmers can gain an ideal yield by using ‘J26’ with no or less N fertilizer in soils with the fertility similar to that in the present study and gain relatively higher output by using ‘X32’, which has high tolerance to abundant N, in more fertile soils.

## Supporting information

S1 File The root yield under different treatments in 2014. (XLS)

S2 File The relevant data in Tables 1–5 and Figs 2 and 3. (XLS)
